# Identification of ADAM12 as a Novel Basigin Sheddase

**DOI:** 10.3390/ijms20081957

**Published:** 2019-04-22

**Authors:** Reidar Albrechtsen, Nicolai J. Wewer Albrechtsen, Sebastian Gnosa, Jeanette Schwarz, Lars Dyrskjøt, Marie Kveiborg

**Affiliations:** 1 Biotech Research and Innovation Centre (BRIC), Faculty of Health and Medical Sciences, University of Copenhagen, 2200 Copenhagen, Denmark; reidar.albrechtsen@bric.ku.dk (R.A.); sebastian.gnosa@bric.ku.dk (S.G.); j.schwarz@ikmb.uni-kiel.de (J.S.); 2 Department of Biomedical Sciences and Department of Clinical Biochemistry, Rigshospitalet, Faculty of Health and Medical Sciences, University of Copenhagen, 2200 Copenhagen, Denmark; nicolai.albrechtsen@sund.ku.dk; 3 Department of Molecular Medicine (MOMA), Aarhus University Hospital, 8200 Aarhus, Denmark; lars@clin.au.dk

**Keywords:** a disintegrin and metalloproteinase, EMMPRIN, CD147, ectodomain shedding

## Abstract

The transmembrane glycoprotein basigin, a member of the immunoglobulin superfamily, stimulates matrix metalloproteinase (MMP)-mediated extracellular matrix (ECM) degradation and thereby drives cancer cell invasion. Basigin is proteolytically shed from the cell surface and high concentrations of soluble basigin in the blood dictates poor prognosis in cancer patients. A positive correlation between basigin and a disintegrin and metalloproteinase (ADAM)-12 in serum from prostate cancer patients has been reported. Yet, the functional relevance of this correlation is unknown. Here, we show that ADAM12 interacts with basigin and cleaves it in the juxtamembrane region. Specifically, overexpression of ADAM12 increases ectodomain shedding of an alkaline phosphatase-tagged basigin reporter protein from the cell surface. Moreover, CRISPR/Cas9-mediated knockout of ADAM12 in human HeLa carcinoma cells results in reduced shedding of the basigin reporter, which can be rescued by ADAM12 re-expression. We detected endogenous basigin fragments, corresponding to the expected size of the ADAM12-generated ectodomain, in conditioned media from ADAM12 expressing cancer cell-lines, as well as serum samples from a healthy pregnant donor and five bladder cancer patients, known to contain high ADAM12 levels. Supporting the cancer relevance of our findings, we identified several cancer-associated mutations in the basigin membrane proximal region. Subsequent in vitro expression showed that some of these mutants are more prone to ADAM12-mediated shedding and that the shed ectodomain can enhance gelatin degradation by cancer cells. In conclusion, we identified ADAM12 as a novel basigin sheddase with a potential implication in cancer.

## 1. Introduction

Basigin (BSG), also named CD147/EMMPRIN, a member of the immunoglobulin family of transmembrane proteins, exerts a wide range of both physiological and pathological functions [1,2,3,4]. Of particular note, BSG regulates the expression and cell surface localization of monocarboxylate transporters-1 (MCT1) and MCT4, allowing the efflux of lactate produced by aerobic glycolysis [5]. Moreover, it induces matrix metalloproteinase (MMP)-mediated extracellular matrix (ECM) degradation, thereby driving tumor invasion and metastasis [6,7,8].

BSG is upregulated in several types of tumors, and soluble BSG concentrations in blood or urine from cancer patients correlate with disease stage and poor prognosis [9,10]. Furthermore, BSG associates with chemotherapy response and survival in bladder cancer [11]. Full-length BSG can be released from tumor cells, macrophages, and stromal cells through micro-vesicle or exosome secretion [3]. However, BSG can also be released into the circulation as a soluble form through proteolytic shedding of its extracellular part (ectodomain). For example, Tang et al. showed a MMP-dependent generation of soluble BSG, lacking the C-terminal part [12], and Egawa et al. demonstrated that MMP14 sheds a 22-kDa N-terminal fragment of BSG into the media of tumor cells [13]. Also, it was recently shown that cholesterol-depletion induces ectodomain shedding of BSG by a disintegrin and metalloproteinase (ADAM)-10 in tumor cells [14].

Like BSG, several of the ADAMs play important roles in cancer. ADAMs are frequently upregulated in human tumors, with high levels of expression indicating worse prognosis [15,16]. Here, ADAM-mediated shedding of membrane bound substrates, such as growth factors, cytokines and adhesion molecules is thought to promote tumor growth and/or progression, best demonstrated by the role of ADAM17 in epidermal growth factor receptor (EGFR) signaling [17]. Another example is ADAM12, which among several substrates is capable of shedding some EGFR ligands [18,19,20] and adhesion molecules like VE-cadherin [21]. Moreover, ADAM12 exerts pro-tumorigenic effects independent of its own protease function [22,23], partly by regulating the activity of MMP14 [24]. A recent study showed a significant correlation between serum levels of ADAM12 and BSG from prostate cancer patients. The two proteins were therefore suggested as biomarker candidates for early diagnosis of prostate cancer [25]. However, the functional relevance of the correlation between ADAM12 and BSG in cancer remains elusive. 

Our present study demonstrates novel functional aspects of the interaction between ADAM12 and BSG. We show that ADAM12 binds BSG and cleaves it in the juxta membrane region, thereby releasing a soluble BSG fragment to the extracellular space. Importantly, assessment of publicly available data revealed a number of cancer-associated BSG mutations in the ADAM12 cleavage region and when tested experimentally, we found that some of the mutations alter the susceptibility to ADAM12-mediated cleavage.

## 2. Results

### 2.1. ADAM12 Interacts with BSG in Human Cells

**Figure 1:**
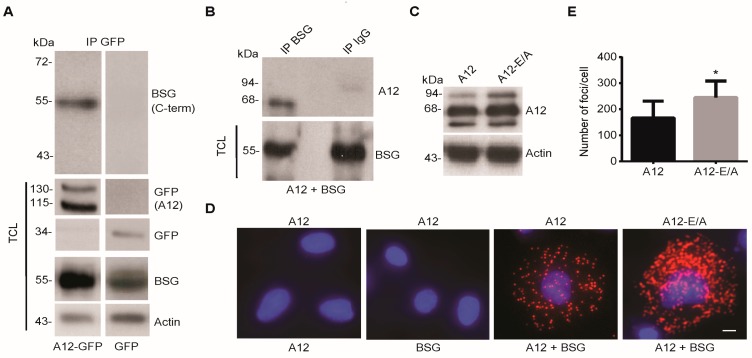
A disintegrin and metalloproteinase (ADAM)-12 interacts with basigin (BSG) in human cells. (**A**) 293-VnR cells were transfected with A12-GFP or GFP alone, immunoprecipitated with a polyclonal anti-GFP antibody and analyzed by Western blot, using an antibody against the C-terminal part of BSG. Total cell lysates (TCL) were tested for A12-GFP (pro and mature forms) and endogenous BSG protein expression. Actin was used as the loading control. (**B**) 293-VnR cells were co-transfected with A12 and full-length BSG, immunoprecipitated with an antibody against BSG and analyzed by Western blot, using a rabbit polyclonal antibody against A12. Immunoprecipitation with control rabbit IgG served as a negative control. (**C**) 293-VnR cells were transfected with A12 or the catalytically inactive A12-E/A mutant, together with full-length BSG and tested for A12 expression by Western blot, using actin as the loading control. (**D**) Cells from C were tested for A12-BSG co-localization by the proximity ligation assay (PLA) (red signal), using antibodies against the extracellular parts of A12 and BSG. Reactions using either of the two antibodies alone served as negative controls. Scale bar = 8 µm. (**E**) Number of red foci were automatically counted in 50 cells from three independent experiments, using MetaMorph microscopy analysis software. The average number of foci per cells ± SEM is shown in the graph. * *p* < 0.05, Student’s *t*-test.

To understand the functional correlation between ADAM12 and BSG, we first examined whether the two proteins interact. To this end, we used 293-VnR cells that express little ADAM12, but substantial amounts of endogenous BSG (Table S1). We transiently expressed ADAM12-Δcyt (hereafter named ADAM12), tagged at the truncated C-terminus with green fluorescence protein (GFP), immunoprecipitated it with an antibody against GFP, and tested it for interaction with endogenous BSG by Western blot. Using an antibody against the C-terminal part of BSG, we detected an approximately 55 kDa protein band in immunoprecipitates from ADAM12-GFP expressing cells, but not from control cells expressing GFP alone (Figure 1A). Performing the reverse co-immunoprecipitation experiment on lysates from cells co-transfected with ADAM12 and full-length BSG, precipitated BSG was capable of pulling down the 68 kDa mature ADAM12 protein, whereas we did not detect ADAM12 when we used control IgG for precipitation (Figure 1B). As the overexpressed ADAM12 protein lacks the cytoplasmic tail, we predict that the interaction site between ADAM12 and BSG resides in the extracellular part of ADAM12. Also, we found that only the mature form of ADAM12 and not the pro form of ADAM12 co-immunoprecipitates with BSG. Since the mature form of ADAM12 is generated by pro protein processing in the secretory pathway [26], this indicates that the interaction between ADAM12 and BSG takes place after transit through the Golgi, possibly at the cell surface.

To examine whether indeed ADAM12 and BSG interact at the cell surface, we used the commercially available Duolink^®^ kit, which is based on the in situ proximity ligation assay (PLA) [27]. We transfected 293-VnR cells with ADAM12 or the catalytically dead ADAM12-E/A mutant (Figure 1C) and stained for ADAM12 and/or BSG. As seen in Figure 1D and quantified in Figure 1E, over-expressed ADAM12 interacts with BSG and the interaction is significantly increased when we express the catalytically inactive ADAM12-E/A mutant. 

### 2.2. ADAM12 Overexpression Increases Ectodomain Shedding of a Truncated BSG Reporter Substrate

**Figure 2:**
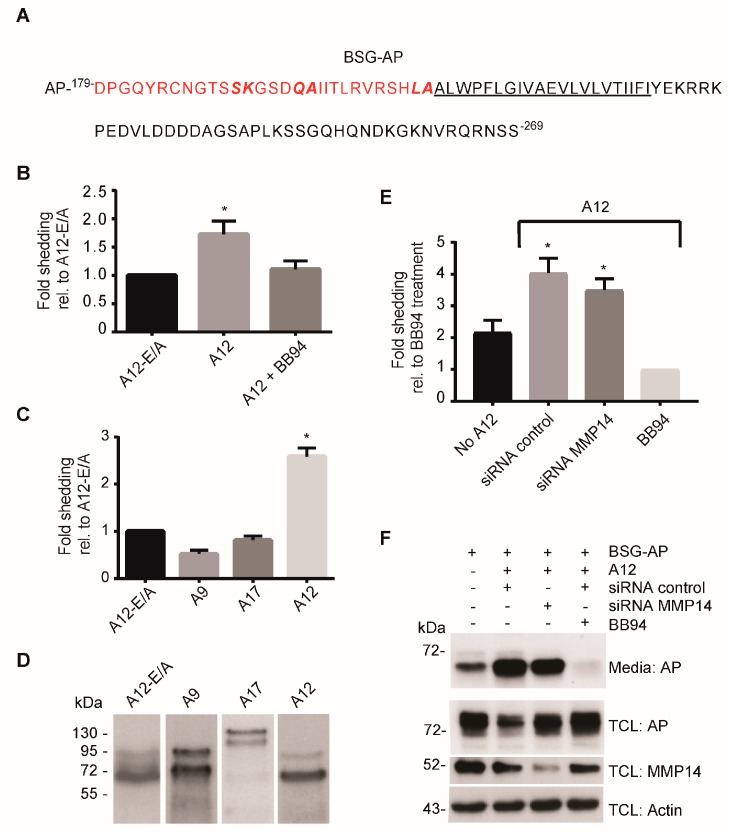
ADAM12 overexpression increases ectodomain shedding of a truncated BSG reporter substrate. (**A**) Amino acid sequence of the reporter substrate BSG-AP, consisting of the C-terminal part of BSG fused to alkaline phosphatase (AP). The truncated extracellular part of BSG is shown in red and the transmembrane domain is underlined. (**B**) Fold shedding in 293-VnR cells transfected with BSG-AP together with A12 or catalytically inactive A12-E/A, and treated with or without the metalloproteinase inhibitor Batimastat (BB94). Fold shedding is calculated as AP activity in the medium divided by the total AP activity in medium and cell lysate, and normalized to A12-E/A. (**C**) Fold shedding in 293-VnR cells transfected with BSG-AP and A12-E/A, A12, ADAM9 (A9) or ADAM17 (A17), calculated as in (**C**). (**D**) Western blot of total lysates from cells used in (**C**), showing comparable expression of the different ADAMs. (**E**) Fold shedding in 293-VnR cells transfected with BSG-AP alone (no A12) or with BSG-AP and A12 together in cells treated with control siRNA, siRNA against matrix metalloproteinase (MMP)-14, or the inhibitor BB94 as indicated. (**F**) Western blot of media and total cell lysates (TCL) from cells in (**E**), using actin as the loading control. For all graphs, values represent means ± SEM from three independent experiments. * *p* < 0.05, ANOVA.

To investigate a potential role of ADAM12 in shedding of BSG, we employed a cell-based shedding assay. The reporter substrate consisted of a truncated form of BSG, containing the extracellular membrane proximal region, the transmembrane domain and the entire cytoplasmic domain fused at the N-terminus to alkaline phosphatase (BSG-AP; Figure 2A). Transfection of 293-VnR cells with the BSG-AP construct, together with either ADAM12 or ADAM12-E/A showed a clear increase in the amount of shed BSG when we expressed the active protease (Figure 2B). Treating the cells with the broad-spectrum metalloproteinase inhibitor Batimastat, known to block ADAM12 activity [21], abrogated the ADAM12-mediated shedding (Figure 2B). 

Demonstrating the specificity of the proteolytic release, BSG-AP shedding was only seen when we overexpressed ADAM12, but not the related ADAM9 or ADAM17 (Figure 2C,D). Since ADAM12 regulates MMP14 [24], which has been previously implicated in the shedding of BSG [12], we tested whether MMP14 was required for the observed ADAM12-mediated shedding of BSG-AP. As expected, since the BSG-AP reporter substrate does not contain the previously reported MMP14 cleavage site [12], knocking down the expression of MMP14 by siRNA did not significantly reduce the increased BSG-AP shedding seen when overexpressing ADAM12 (Figure 2E). Complementing these shedding data, Western blot analysis of the conditioned media, using an antibody against alkaline phosphatase (AP), showed a band corresponding to the shed AP-fusion protein, which had approximately the same intensity when analyzing media from MMP14 and control siRNA transfected cells, whereas media from cells treated with Batimastat showed little to no shed protein (Figure 2F, top lane). Of note, the shedding assay showed a certain level of BSG-AP shedding in the absence of exogenous ADAM12 expression (Figure 2E), which we could block with the protease inhibitor Batimastat. This shedding activity may reflect the expression of other sheddases in 293-VnR cells. 

### 2.3. CRISPR/Cas9-Mediated ADAM12 Knockout Reduces BSG Reporter Shedding

**Figure 3:**
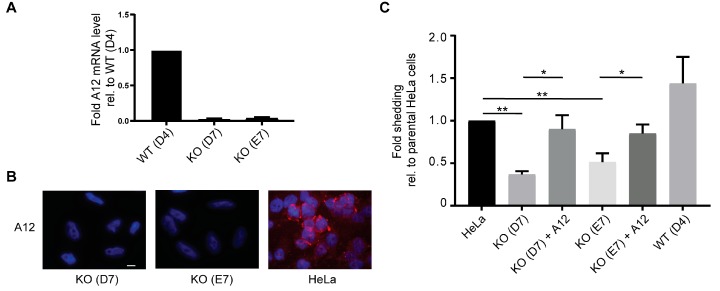
CRISPR/Cas9-mediated ADAM12 knockout reduces BSG reporter shedding. (**A**) qPCR analysis of A12 mRNA in CRISPR/Cas9 generated A12 knockout (clones D7 and E7) and non-edited wildtype (clone D4) HeLa cells. (**B**) Immunofluorescence staining of A12 in CRISPR/Cas9 generated A12 knockout HeLa clones (KO) D7 and E7, and parental HeLa cells. Nuclei were stained with 4′,6-diamidino-2-phenylindole (DAPI) and scale bar = 12 μm. (**C**) Fold shedding of BSG-AP transfected parental HeLa cells, CRISPR/Cas9-generated A12 knockout (KO) HeLa clones D7 or E7 with or without A12 re-expressed, and the non-edited wildtype (WT) clone D4, as indicated. Values represent means ± SEM from three independent experiments. * *p* < 0.05, ** *p* < 0.005, ANOVA.

To corroborate the importance of ADAM12 for shedding of BSG, we knocked out ADAM12 expression in HeLa cells, known to express endogenous ADAM12 (Table S1), using CRISPR/Cas9 gene editing. Based on sequencing of established cell clones, we chose the knockout clone D7, which harbors deletions of 4 and 14 nucleotides, and the E7 clone that harbors 4 nucleotides deletion on both alleles. We used parental HeLa cells and the non-edited clone D4 as control cell lines. We validated the lack of ADAM12 expression in D7 and E7 cell lines by qPCR analysis (Figure 3A) and immunofluorescence staining (Figure 3B). When we tested clones D7 and E7 for BSG-AP shedding, we observed a decreased shedding for both ADAM12 knockout clones, as compared to parental HeLa cells or the D4 control cell line (Figure 3C). Importantly, re-expressing ADAM12 in both D7 and E7 cells partially rescued the lack of BSG-AP shedding (Figure 3C). 

### 2.4. ADAM12 Sheds Endogenous BSG

**Figure 4:**
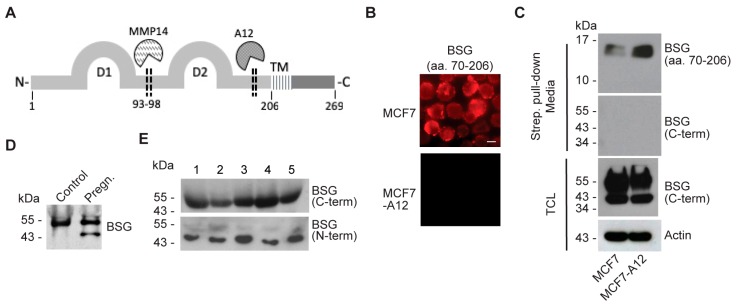
ADAM12 sheds endogenous BSG. (**A**) Schematic of the BSG structure, showing the two immunoglobulin domains (D1 and D2), the identified MMP14 and putative ADAM12 (A12) cleavage sites, and the transmembrane domain (TM). (**B**) Cytospin of wildtype or A12 expressing MCF7 cells were immuno-stained with the antibody PA5-29787, recognizing region aa. 70–206 of BSG. Scale bar = 8 µm. (**C**) Wildtype or A12 expressing MCF7 cells were surface biotinylated and incubated overnight in serum free media. Biotinylated cell surface proteins were pulled down with streptavidin–agarose from conditioned cell media or total cell lysates (TCL) and detected by Western blot, using antibodies against aa. 70–206 (PA5-29787) or the C-terminus of BSG. Actin was used as a loading control. (**D**) Western blot of BSG in serum from pregnant (pregn.) or non-pregnant (control) women. (**E**) Western blot of BSG in serum from five bladder cancer patients stained with antibodies recognizing either the C- or the N-terminus of BSG.

To assess ADAM12-mediated shedding of full-length endogenous BSG, we stably expressed ADAM12 in MCF7 human breast cancer cells, which express no detectable endogenous ADAM12 (Table S1). To detect the shed BSG ectodomain, we used an antibody recognizing a peptide (amino acids 70–206) located between the known MMP14 cleavage site and the region suggested to be cleaved by ADAM12 (Figure 4A). Immunofluorescence staining, using this antibody, detected a substantial amount of endogenous BSG at the cell surface of most MCF7 cells (Figure 4B). In contrast, the fluorescence signal was almost completely absent in MCF7 cells expressing ADAM12 (Figure 4B), indicating that the BSG ectodomain was shed from these cells. Also, using cell surface biotinylation and streptavidin pull-down of biotinylated proteins, we could detect more of the endogenous BSG ectodomain by Western blot in media from ADAM12 expressing MCF7 cells, as compared to wildtype MCF7 cells (Figure 4C). Supporting the notion that the soluble BSG protein is proteolytically shed from the cell surface rather than secreted (e.g., in exosomes), we did not detect any bands in conditioned media when blotting with an antibody against the intracellular C-terminal part of BSG (Figure 4C). In contrast, bands corresponding to full-length BSG and the cleaved C-terminal fragment were identified in total cell lysates when using this antibody (Figure 4C). 

Human placenta produces high amounts of ADAM12, both the transmembrane ADAM12-L and the soluble ADAM12-S isoforms [28]. To assess the physiological relevance of ADAM12-mediated BSG shedding, we examined whether soluble BSG could be detected in serum from pregnant women. As shown by Western blot analysis, we detected a 55 kDa band, corresponding to full-length BSG, in pregnant or non-pregnant women, whereas we could only detect an approximately 48 kDa band in serum from pregnant women (Figure 4D). Similarly, we detected bands of a size corresponding to shed BSG in a Western blot of sera from five bladder cancer patients (Figure 4E), which we previously reported exhibit high levels of soluble ADAM12 in the circulation [29]. 

### 2.5. Cancer-Associated BSG Mutants Are Differentially Shed by ADAM12

**Figure 5:**
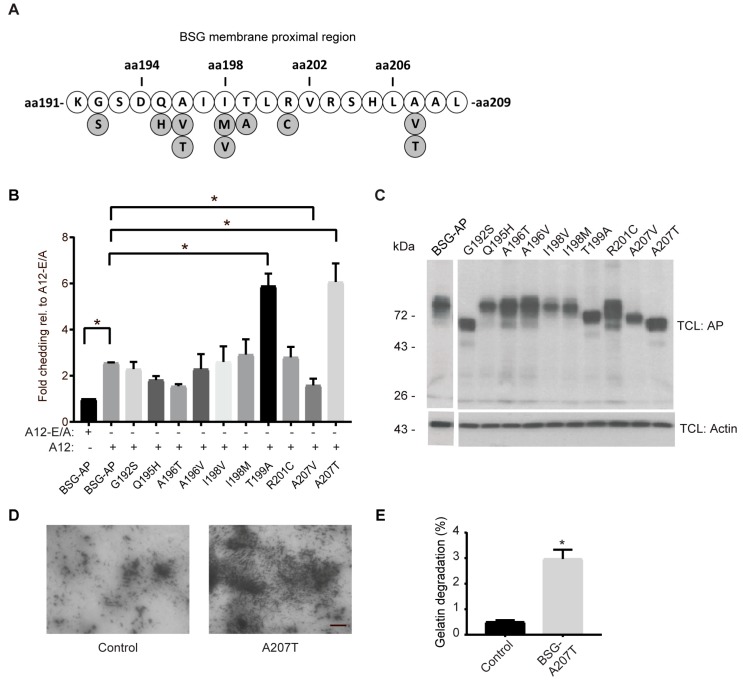
Cancer-associated BSG mutants are differentially shed by ADAM12. (**A**) Illustration of cancer-associated mutations (marked in grey) in the extracellular membrane proximal region (aa. 190–208) of BSG, identified in the Catalogue Of Somatic Mutations In Cancer (COSMIC) database (BSG, ENST00000333511; August 2017). (**B**) Fold shedding in 293-VnR cells transfected with A12 or the catalytically inactive A12-E/A mutant together with wildtype (BSG-AP), or various BSG-AP point mutations identified in (**A**). (**C**) Western blot of AP in total cell lysates (TCL) from cells in (**B**). Actin was used as loading control. (**D**) In situ gelatinase assay, using HT1080 cells grown on Oregon green-labeled gelatin and treated with conditioned media from untransfected 293-VnR cells (control) or 293-VnR cells transfected with ADAM12 together with a full-length BSG expression construct harboring the A207T mutation. Dark areas (without green fluorescence) represent gelatin degradation, scale bar = 10 µm. (**E**) Gelatin degradation was measured in µm^2^ for 27 images, using MetaMorph software and the percentage degradation was calculated. For both graphs, values represent means ± SEM from three independent experiments. * *p* < 0.05, Student’s *t*-test or ANOVA.

When examining the extracellular membrane proximal region of BSG that is cleaved by ADAM12 (marked by red in the BSG-AP reporter substrate in Figure 2A), we noticed three amino acid pairs (SK, QA, LA), which were also found in the cleavage site of tumor necrosis factor (TNF)-α and constitutes the quenched fluorescence peptide substrate frequently used for measuring the catalytic activity of several ADAMs, including ADAM12 [30]. Searching the COSMIC (Catalogue Of Somatic Mutations In Cancer) database for mutations in the BSG gene associated with a broad range of cancers (https://cancer.sanger.ac.uk/cosmic), there were several somatic mutations in the part encoding this extracellular membrane proximal region (Figure 5A). To investigate the consequence of these mutations for BSG shedding, we introduced the identified individual single point mutations in the BSG-AP construct, co-transfected the constructs together with ADAM12, and performed shedding assays. Of the ten mutations tested, two of the cancer-associated mutations (T199A and A207T) were more efficiently shed compared to the control wildtype construct, whereas one mutation (A207V) resulted in significantly reduced shedding (Figure 5B). Notably, the BSG-AP mutants were expressed to approximately similar levels, but ran at slightly variable molecular weights, as shown by Western blot analysis (Figure 5C), indicating that some of the mutations changed the protein glycosylation pattern.

The biological role of BSG shedding is not fully understood; yet, several studies have shown a stimulatory effect of the MMP14-generated BSG ectodomain on MMP activation, particularly activation of the secreted MMP2 collagenase [2,3,8]. To test the effect of the ADAM12-generated ectodomain on collagen degradation, we performed an in situ gelatinase assay [24]. Specifically, we added conditioned media from control 293-VnR cells or cells expressing ADAM12, together with the shedding prone BSG-A207T mutant, onto HT1080 cells grown on Oregon green gelatin and incubated the cells for 24 h. Little gelatin degradation was observed when media from control cells was added to the HT1080 cells (Figure 5D); however, gelatin degradation was clearly enhanced when we added media from BSG-A207T expressing cells to the HT1080 cells (Figure 5D).

## 3. Discussion

The proteolytic shedding of BSG from the cell surface is well described [12,13,14]. However, our data are the first to demonstrate that, in addition to MMP14 and most recently ADAM10, also ADAM12 is capable of shedding BSG. By ADAM12 overexpression and siRNA- or CRISPR-Cas9-mediated ADAM12 depletion, we demonstrate that the transmembrane form of ADAM12 cleaves BSG in the extracellular membrane proximal region. In contrast, we observed no BSG shedding when overexpressing the shorter secreted ADAM12 isoform. While MMP14 has been shown to cleave BSG between the two immunoglobulin-like domains IgC2 (D1: aa. 25–101) and IgI (D2: aa. 106–200), generating a 22 kDa soluble fragment [13], we showed that ADAM12 generates an approximately 48 kDa ectodomain, containing both immunoglobulin-like domains (Figure 4A). The protein region harboring the ADAM12 cleavage site contains three amino acid pairs (SK, QA, LA), which were previously referred to by Moss et al. as the potential cleavage site found in a short peptide of TNFα, used to measure the catalytic activity of multiple MMPs and ADAMs [30]. While this suggests that other MMPs or ADAMs would likely also be able to cleave within this region, our data indicate that neither MMP14, nor ADAM9 or ADAM17 cleave at this site. As for ADAM10-mediated BSG shedding, the published study focused on the intracellular cytoplasmic fragment generated by intramembranous proteolysis following the initial extracellular cleavage event and did not mention the size of the shed ectodomain [14]. 

According to previous reports, the generated soluble BSG ectodomain acts to stimulate the activity of MMPs [12,13,31,32,33], for which reason BSG is also designated EMMPRIN—extracellular matrix metalloprotease inducer [6]. For example, it appears that soluble BSG, shed from the cell surface of cancer cells, acts in a paracrine manner to stimulate the expression of MMP2 in the surrounding stromal fibroblast [31]. Furthermore, it has been suggested that membrane bound BSG on fibroblasts functions as a receptor for the soluble BSG ectodomain, which upon binding gets internalized and activates the ERK1/2 signaling pathway, thereby inducing the expression of MMP1, MMP2, and MMP3 [34]. Based on our findings, the soluble BSG fragment generated by ADAM12 can increase gelatinase activity when added to cancer cells in vitro. In this context, it is worth mentioning that ADAM12 has been previously shown to enhance the gelatinase activity of MMP14, through a mechanism involving a protein complex composed of ADAM12, MMP14, and the adhesion receptor α_v_β_3_ [24]. Thus, it appears that ADAM12 contributes to BSG shedding and MMP activation both directly and indirectly by enhancing MMP14 activity.

To assess the relevance of our in vitro findings, we examined serum samples from pregnant versus non-pregnant healthy women, as well as five bladder cancer patients by Western blot. Here we detected a protein band, most likely corresponding to full-length BSG released in exosomes. Moreover, we detected a smaller protein band of approximately 48 kDa in all cancer samples, as well as in pregnancy serum, but not in the non-pregnancy serum. Based on the band size and the fact that we only detected the band when we used an antibody against the N-terminal extracellular part, but not an antibody binding in the intracellular C-terminal part, this could potentially represent the ADAM12 generated BSG ectodomain. Supporting this idea, we previously reported that ADAM12 is highly upregulated in the same serum samples where we detected the 48 kDa band [29]. Thus, as previously suggested for ADAM12, it could be interesting to investigate if presence of this BSG fragment in plasma samples constitutes a potential prognostic marker. 

In line with the shed BSG ectodomain being a potential biomarker in cancer, we found a number of cancer-associated mutations in the region harboring the ADAM12 cleavage site. When introducing the individual mutations in the exogenous BSG-AP substrate, we found two of the mutations (T199A and A207T) to be more susceptible to ADAM12-mediated shedding. Also, one of these BSG mutations (T199A) appeared to change the size of the shed BSG fragment, thereby potentially shifting the ADAM12 cleavage site. Indeed, some variability of the cleavage site has been reported for the shedding of BSG by MMP14 [13]. Looking into the structural information, BSG has been shown to function as a dimer [34], and the dimerization of the two BSG molecules were suggested to take place around amino acids 184–196 [35], which is exactly at the beginning of the region harboring the identified cancer mutations. While our data does not reveal any effects of BSG dimerization on shedding efficiency, our findings provide an example of how proteolytic events can be affected not only at the level of the protease, but also by altering the susceptibility of the substrate for being cleaved under particular pathological conditions. 

In conclusion, the present study provides mechanistic insight on the pro-tumorigenic role of ADAM12 in cancer. ADAM12-mediated shedding of BSG could promote tumor progression by stimulating MMP activation and enabling cancer cell invasion.

## 4. Materials and Methods 

### 4.1. Antibodies and Reagents

Antibodies against ADAM12 were as previously described [23,36,37]. The rabbit polyclonal antibodies N-19 (sc-9752) and H-200 (sc-13976) against BSG, and goat polyclonal antibody L-19 against PLAP (sc-9757) were from Santa Cruz Biotechnology (Dallas, TX, USA). Other antibodies used were rabbit polyclonal antibody PA5-29787 against BSG (Thermo Fischer Scientific, Waltham, MA, USA), mouse monoclonal antibodies against actin (MAB1501, Millipore Chemicon, Burlington, MA, USA) and FITC-conjugated anti-CD147 (555962, BD Biosciences, San Jose, CA, USA). Living Colors^®^ GFP Monoclonal Antibody from Clontech was used for co-immunoprecipitation studies. Secondary antibodies used were horseradish peroxidase (HRP)-conjugated goat anti-mouse, goat anti-rabbit, and rabbit anti-goat immunoglobulins from DAKO A/S (Glostrup, Denmark). Alexa Fluor 488-conjugated rabbit anti-goat IgG and goat anti-mouse IgG, and Alexa Fluor 546-conjugated goat anti-mouse IgG F(ab)_2_ fragment and goat anti-rabbit IgG F(ab)_2_ fragment were from Invitrogen (Taastrup, Denmark). The metalloprotease inhibitors Batimastat, GM6001, and all other chemicals were from Merck KGaA (Darmstadt, Germany).

### 4.2. Plasmid

Mammalian expression constructs encoding full-length human ADAM12-L (ADAM12), human ADAM12-L fused to GFP (ADAM12-GFP), or human ADAM12-L lacking the cytoplasmic tail (ADAM12-Δcyt) were as previously described [24]. A point mutation in the catalytic site (E351Q) of ADAM12 (ADAM12-E/A) was introduced using the Phusion Site-directed mutagenesis kit (Thermo Fischer Scientific, Waltham, MA, USA). For retroviral transduction, cDNA encoding ADAM12 and ADAM12-E/A was cloned into the retroviral expression vector pRevTRE (Clontech, BD Biosciences). Full-length human BSG isoform 2 (OHu27639) cDNA was obtained from GeneScript (Piscataway, NJ, USA) and inserted into the pcDNA3.1 vector. The expression construct encoding alkaline phosphatase (AP)-tagged BSG (BSG-AP) was provided by Carl Blobel (New York, NY, USA), and have been described previously [21]. Using a PCR-based method [38], single point mutations were introduced into the BSG-AP construct or the full-length BSG construct as indicated. ADAM9 and ADAM17 expression constructs were from William English (Sheffield, UK), and Gillian Murphy (Cambridge, UK), respectively.

### 4.3. Cell Culture and Transfections

The HEK293 cell line stably expressing the vitronectin receptor αVβ3 integrin, called 293-VnR, was previously described [39]. The cancer cell lines MCF-7, HeLa, and HT1080 were from ATCC (LGC Standards AB, Boras, Sweden) and cultured as previously described [36]. All cell lines were transiently transfected using X-tremeGENE9 Transfection Reagent (Roche Applied Science, Hvidovre, Denmark). MCF-7 cells stably expressing ADAM12-Δcyt in a tetracycline (tet)-Off system (MCF7-A12) were generated by retroviral transduction of ADAM12-Δcyt in the pRevTRE vector (Clontech, BD Sciences) as described previously [40]. The MCF7-A12 cell line was kept in growth medium supplied with 50 mg/mL hygromycin B (Roche Applied Science) and 100 mg/mL geneticin (Sigma-Aldrich, St. Louis, MO, USA). Small interfering RNAs (siRNAs) against MMP14 were obtained as siGENOME SMARTpool reagents from Thermo Scientific (Dharmacon, Waltham, MA, USA), and siRNA universal negative control was from Sigma-Aldrich. siRNA transfection was performed according to the manufacturer’s instructions, using OPTI-MEM I and Lipofectamine^TM^ 2000 (Invitrogen). 

### 4.4. CRISPR/Cas9 Gene Editing

HeLa ADAM12 knockout cells were generated using the CRISPR-Cas9 system. Guide RNAs (gRNA) were designed using the WTSI genome editing tool [41] and individually inserted in the vector pSpCas9(BB)-2A-GFP [42]. To determine the gRNA editing efficiency, HeLa cells were transfected with the pSpCas9(sgRNA)-2A-GFP vectors and verified by Indel Detection by Amplicon Analysis (IDAA). gRNA 5-TACCGTGTAATTTCGAGCGA-3, targeting exon 4, showed the highest editing efficiency and was subsequently transfected into HeLa cells. GFP positive cells were single cell sorted, expanded and tested by qPCR and Western blot for ADAM12 knockout. Additionally, we screened positive clones for biallelic frameshifts using Sanger sequencing. Two individual ADAM12 knockout clones, D7 and E7, as well as one non-edited clone D4, were used for further experiments.

### 4.5. Human Serum Samples

Control and pregnancy serum samples obtained from healthy individuals and serum samples from patients with bladder carcinomas were obtained as previously described [29]. Informed consent was obtained from all patients and the bladder cancer protocol was approved by The National Committee on Health Research Ethics (#1708266).

### 4.6. Shedding Assay

ADAM12-mediated shedding of AP-tagged substrates was determined as described previously [21]. The shedding activity was calculated as AP activity in conditioned medium divided by AP activity in the medium plus corresponding cell lysate after subtracting the background signal from non-transfected cells. The value from cells transfected with the AP construct alone or together with ADAM12-E/A was set to 1. Western blot analysis of total cell lysates was performed to ensure equal expression of the constructs.

### 4.7. Immunofluorescence Staining

Visualization of ADAM12 and BSG by immunofluorescence staining was performed using standard techniques as previously described [36]. Cell surface staining of BSG was done using a cytospin method, as described by Kawaguchi et al. [43]. In brief, cells were trypsinized and stained without permeabilization, fixed in 4% paraformaldehyde, and spun down in a cytospin centrifuge (Sandon, Thermo Fisher Scientific, Waltham, MA, USA). For staining of adherent cells, fixation by either cold methanol or 4% paraformaldehyde was used. BSG-stained cells were counted and the total grey value was measured at 546 nm using MetaMorph software and a multi wavelength cell-scoring program. For visualization of protein co-localizing at the cell surface, Duolink reagents from Olink (Uppsala, Sweden) were used to stain non-permeabilized cells, as recently described [26]. Fluorescence imaging was performed using an inverted Zeiss Axiovert 220 Apotome system equipped with a 63/1.4 Plan-Apochromat water immersion objective. The images were processed using the Axiovision program (Carl Zeiss, Oberkochen, Germany) and MetaMorph software. Average number of foci from 50 cells from ADAM12 or ADAM12-E/A transfected cells were compared.

### 4.8. In Situ Gelatinase Assay

The in situ gelatinase assay was performed as previously described [24]. In brief, cells were seeded on 3.5 cm dishes coated with gelatin (10 mg/mL) coupled to Oregon green 488 dye (G-13186) from Molecular Probes (Life Technologies, Taastrup, Denmark). Twenty-four hours after, gelatin degradation was quantified by measuring the area of black holes in the fluorescent gelatin relative to the total area, using MetaMorph software (MM45, Molecular Devices, San Jose, CA, USA). 

### 4.9. Immunoprecipitation and Western Blot Analysis

Protein immunoprecipitation and Western blot analysis were performed using standard protocols, as previously described [21,24]. In brief, 293-VnR cells transfected with ADAM12-GFP or with basigin were extracted in RIBA buffer for 20 min, containing inhibitors as described [20,23]. The extracts were incubated with antibodies for 2 h at 4 °C with gentle agitation. Protein G-SepharoseTM 4 Fast Flow beads (GE Healthcare, Chicago, IL, USA) were added for an additional 1 h at 4 °C. Beads were gently washed three times in RIPA buffer (20 mM Tris-HCl (pH 7.5) 150 mM NaCl, 1 mM Na2EDTA, 1% Triton X-100). Bound proteins were eluted in 2× sample buffer, followed by Western blot analysis. Cell surface proteins were biotinylated using non-cleavable EZ-Link Sulfo-NHS-LC-Biotin, pulled down with streptavidin–agarose, and analyzed by Western blotting as previously described [44].

### 4.10. Quantitative PCR

Total RNA extraction was performed using GeneJet RNA Purification kit (Thermo Scientific), cDNA synthesis, and quantitative PCR (qPCR) and was carried out as described earlier [21]. They were performed with ADAM12 primers 5-CAGGCACAAAGTGTGCAGAT-3, 5-GCTTGTGCTTCCTCCAAAGC-3 and BSG primers 5-GACGTCCTGGATGATGACGA-3, 5-GAAGAGTTCCTCTGGCGGAC-3. The gene for ribosomal phosphoprotein (RPO) was used as a house keeping reference gene, and RPO primers 5-CAGCAGTTTCTCCAGAGC-3, 5-TTCATTGTGGGAGCAGAC-3, and data were analyzed using the 2(−ΔΔ*C*_T_) method. 

### 4.11. Statistical Analysis

Statistical analyses of all experiments were performed for three independent repeats using Student’s *t* test for comparing two groups and ANOVA for multiple group comparisons; *p* < 0.05 was considered statistically significant.

## Supplementary Materials

Supplementary materials can be found at https://www.mdpi.com/1422-0067/20/8/1957/s1.

## Abbreviations

ADAM	A disintegrin and metalloproteinase
AP	Alkaline phosphatase
BSG	Basigin
COSMIC	Catalogue of somatic mutations in cancer
ECM	Extracellular matrix
EGFR	Epidermal growth factor receptor
EMMPRIN	extracellular matrix metalloprotease inducer
HRP	Horseradish peroxidase
MCT	Monocarboxylate transporter
MMP	Matrix metalloproteinase
PLA	Proximity ligation assay
TNFα	Tumor necrosis factor alpha
