# Supplementary material for: Identification of ADAM12 as a Novel Basigin Sheddase
Source: Int J Mol Sci. 2019 Apr 22;20(8):1957. doi: 10.3390/ijms20081957 (PMC6514901; doi:10.3390/ijms20081957)
Supplement: Supplementary file 1 [file ijms-20-01957-s001.pdf]

Identification of ADAM12 as A Novel Basigin Sheddase

Reidar Albrechtsen, Nicolai J. Wewer Albrechtsen, Sebastian Gnosa, Jeanette Schwarz, Lars Dyrskjøt and Marie Kveiborg

Supplementary table 1:

	**HEK293**	**HeLa**	**MCF-7**
ADAM12	0,5	17,4	0,0
ADAM10	19,8	25,3	13,4
Basigin	765,4	460,8	894,4

RNAseq transcript per million (www.proteinatlas.org)
